# The Medicine Revolution Through Artificial Intelligence: Ethical Challenges of Machine Learning Algorithms in Decision-Making

**DOI:** 10.7759/cureus.69405

**Published:** 2024-09-14

**Authors:** Marta Marques, Ana Almeida, Helder Pereira

**Affiliations:** 1 Anesthesiology, Centro Hospitalar Universitário São João, Porto, PRT; 2 Surgery and Physiology, Faculty of Medicine, Universidade do Porto, Porto, PRT

**Keywords:** artificial intelligence in medicine, ethic consideration, evidence base medicine, machine learning in medicine, meical ethics

## Abstract

The integration of artificial intelligence (AI) and its autonomous learning processes (or machine learning) in medicine has revolutionized the global health landscape, providing faster and more accurate diagnoses, personalization of medical treatment, and efficient management of clinical information. However, this transformation is not without ethical challenges, which require a comprehensive and responsible approach. There are many fields where AI and medicine intersect, such as health education, patient-doctor interface, data management, diagnosis, intervention, and decision-making processes. For some of these fields, there are some guidelines to regulate them. AI has numerous applications in medicine, including medical imaging analysis, diagnosis, predictive analytics for patient outcomes, drug discovery and development, virtual health assistants, and remote patient monitoring. It is also used in robotic surgery, clinical decision support systems, AI-powered chatbots for triage, administrative workflow automation, and treatment recommendations. Despite numerous applications, there are several problems related to the use of AI identified in the literature in general and in medicine in particular. These problems are data privacy and security, bias and discrimination, lack of transparency (Black Box Problem), integration with existing systems, cost and accessibility disparities, risk of overconfidence in AI, technical limitations, accountability for AI errors, algorithmic interpretability, data standardization issues, unemployment, and challenges in clinical validation. Of the various problems already identified, the most worrying are data bias, the black box phenomenon, questions about data privacy, responsibility for decision-making, security issues for the human species, and technological unemployment. There are still several ethical problems associated with the use of AI autonomous learning algorithms, namely epistemic, normative, and comprehensive ethical problems (overarching). Addressing all these issues is crucial to ensure that the use of AI in healthcare is implemented ethically and responsibly, providing benefits to populations without compromising fundamental values. Ongoing dialogue between healthcare providers and the industry, the establishment of ethical guidelines and regulations, and considering not only current ethical dilemmas but also future perspectives are fundamental points for the application of AI to medical practice. The purpose of this review is to discuss the ethical issues of AI algorithms used mainly in data management, diagnosis, intervention, and decision-making processes.

## Introduction and background

The term “artificial intelligence” (AI), first used by John McCarthy in 1956, refers to the capacity of a machine or software to simulate intelligent human behavior to perform instantaneous calculations, solve problems, and evaluate new data based on previously analyzed information [1-3]. AI systems are designed to learn, reason, perceive their surroundings, and make autonomous decisions. Using complex algorithms and mathematical models, AI has the potential to process large volumes of data, identify patterns, and improve its performance over time. The applications of AI are vast, ranging from speech recognition and computer vision to advanced medical diagnostics, industrial automation, and virtual assistants [1,3]. The integration of AI in medicine represents a milestone. Among the various well-developed areas of AI, one that stands out in the field of medicine is the capacity for autonomous learning, or machine learning (ML). Although they are interrelated, they are not the same: while AI is a broad field of computer science focused on creating systems that can perform tasks typically requiring human intelligence, autonomous learning, or ML, is a subcategory of AI that specifically focuses on developing algorithms and techniques that allow computers to learn from data and improve their performance on specific tasks over time without being explicitly programmed to do so [3-7].

Instead of following predefined instructions, autonomous learning systems are designed to learn from data. There are different approaches and techniques within autonomous learning, but the fundamental idea is to enable a system to improve its performance as it is exposed to more data (concepts of self-improvement and self-learning) [8]. There are different types of autonomous learning: supervised learning, unsupervised learning, reinforcement learning, and transfer learning [9]. The supervised learning method is a training model that uses a labeled dataset, where each input is associated with a desired output. The “inputs” refer to the information provided to the model, while the “outputs” are the corresponding responses [4]. The algorithm learns to establish a relationship between these inputs and outputs during the “training” process, allowing the model to make predictions or decisions when exposed to a new dataset based on the learned pattern. The unsupervised learning method consists of the unlabeled dataset, where there are no outputs associated with the inputs; the algorithm attempts to find patterns in the data on its own. The reinforcement learning method is a model where the AI “learns” through interaction with an environment: the agent takes actions in an environment and receives rewards or penalties in response (like Pavlov’s classical conditioning) [10]. Finally, the transfer learning method is an approach that combines elements of both supervised and unsupervised learning for situations where both labeled and unlabeled data are available [4].

These ML algorithms are applied in a wide variety of medical fields, including disease pattern recognition, clinical data processing, medical diagnosis, therapeutic recommendation, and much more [6]. However, the increasing use and application of ML algorithms, especially in the field of medicine, has raised a series of ethical questions: it becomes crucial to enumerate the most pertinent ethical issues arising from the use of AI and ML algorithms in medicine [11].

## Review

Methodology

To carry out this narrative review of the literature, some steps were taken. We searched scientific literature published in the indexed databases PubMed and Embase, using the following keywords: artificial intelligence (AI), ethics, medicine, machine learning, epistemic considerations, normative considerations, comprehensive considerations, and ethical considerations. Abstracts of obtained articles were reviewed to ensure their relevance to the research question. The selected studies were then analyzed, integrating the information into the review as appropriate.

Ethical considerations regarding the use of AI in medicine

The application of AI in healthcare brings a series of ethical issues, and it is important to address these concerns to ensure the responsible use of this technology. Among the various ethical problems identified in the literature on the use of AI in general, and in medicine in particular, the most widely discussed are data bias, lack of transparency, the black box phenomenon, issues regarding data privacy, responsibility for decision-making, security concerns for the human species, and technological unemployment [12].

Data Bias

This occurs if the information used to train an AI model reflects biases already present in society. AI algorithms will be only as good as the data used in the learning process. However, since these data are produced by humans, their bias is inevitable [13]. As a consequence, the model will reproduce these biases, leading to decisions made by the model becoming discriminatory and/or unjust [13]. Recently, critical areas where data bias most significantly affects the AI decision-making process were identified, particularly in the field of medical coding and patient health data management [12]. To overcome data bias in AI decision-making algorithms, it is important to ensure that the data used to train AI are representative of all relevant demographic groups; this mitigates the risk of non-representation. Additionally, regular auditing and monitoring of AI systems are essential to detect and correct bias throughout the model development and deployment process. Algorithmic transparency, allowing the interpretation of decision-making mechanisms, is also fundamental, as it allows the identification and resolution of biased decisions [12,13]. In the specific context of medical coding and health data management, additional layers of preventative measures can help mitigate bias. These measures are the following: ensuring that medical coding systems are standardized and representative of the population; integrating experts at different points in the algorithm design process to ensure that AI’s understanding of health data reflects a variety of perspectives; and monitoring the decision-making process based on AI data (along with feedback loops from healthcare providers) to identify areas where the system may be introducing bias.

Transparency

The lack of transparency in AI is one of the most complex issues to address [14,15]. In various fields, including healthcare, transparency requirements are crucial as the decisions directly affect people's lives. Transparency should be achieved through a set of measures such as interpretability and explainability, communication, auditability, traceability, provision of information, record-keeping, data governance, and documentation [15,16], such as defined in a European Union (EU) directive recently published [16]. In this scope, it is easy to anticipate that, in certain ML models, especially in complex models such as neural networks, this definition of transparency is difficult to achieve (the results are impossible to reproduce due to ignorance of the decision algorithm) [13-15].

Black Box Phenomenon [17]

Feared for their complexity and lack of transparency, the algorithms and tools used by the “black box” (or “brain”) of AI present an obstacle to the process of human understanding and subsequent certification [5,18]. Currently, it is impossible for humans to understand how AI reached a particular conclusion, which is considered correct. Since the algorithms are not humanly understood, adaptation to governance (human control) is compromised [19]. To mitigate this “black box phenomenon,” the continuous assessment of the safety and efficacy of the tools and algorithms used by the “black box” of AI becomes mandatory [17,19]. If AI achieves full autonomy, these “phenomena” will become so frequent and incomprehensible that any attempt at regulation by current governance systems will be impossible [18]. According to the EU directive, all AI systems should be lawful, ethical, and robust: AI must respect all applicable laws and regulations and also ethical principles and values; and AI must be reliable from a technical point of view, minimizing its impact and interaction with the social environment [16].

Data Privacy

In an era where data protection is essential, the process of teaching (“teaching machines to learn”) becomes challenging. It is crucial to ensure the security and confidentiality of the data used by the AI at various levels: regarding the companies owning the AI patent, and, perhaps most importantly, regarding third parties, namely, competing companies or “hackers” [20]. EU regulation requires AI developers to incorporate data minimization into their products, stating that data collection should mirror reasonable expectations and that only data strictly necessary for the specific context is collected. In addition, it recognizes the role of individual rights and user control over the collection, processing, transfer, and deletion of personal data (in accordance with legal principles within the General Data Protection Regulation) [21].

Responsibility in Decision-Making

AI makes decisions based on algorithms without human intervention in the process. It is up to the healthcare professional to validate this decision. However, healthcare professionals do not have an active part in the decision-making process, and, frequently, they do not know how the decision or result was achieved by the AI (as seen in the issue of the black box phenomenon) [3,17]. All AI decisions, even the quickest, carry responsibility and moral consequences, not for the machine itself but for its creators and users [22,23]. The question is not whether healthcare professionals were aware of the potential risks and bias in decision-making, but whether they had the ability to understand these risks and bias [22]. The primary ethical and legal questions are the following: if something goes wrong in the decision-making process (i.e., decisions that harm the patient), should the blame be solely on the healthcare professional who merely validated the AI’s decision? If the professional is not the only one responsible, how can responsibility be attributed to an entity that is currently not a legal person, such as AI? In this case, can or should the responsibility fall on the creators of the AI? [3,13,23].

AI Safety for the Human Species

AI is a machine capable of executing a complex range of tasks that humans can control at the beginning. However, with the ability to learn and improve its own code (e.g., ML capabilities), AI has the potential to enhance its self-improvement capacity, learn how to bypass any restrictions in its code, and develop its own purposes [24]. Intentional or not, a general-purpose machine capable of self-improvement or self-learning with superior intelligence and performance across various dimensions could have, hypothetically, serious impacts on human beings [24]. The dimensions in which machines could exhibit superior intelligence are learning and adaptability (ability to improve and adapt over time through experience or new data), efficiency (speed and effectiveness in performing tasks compared to humans), creativity (capacity to generate new ideas or solutions), accuracy (precision in executing tasks or making predictions), and autonomy (degree of independence in operating and making decisions without human intervention).

Technological Unemployment

As AI and, consequently, robotics evolve, there are some ethical concerns about the impact on future employability. The economic model prevalent in most countries is based on compensation in exchange for a task (profession) performed. Currently, it is a challenge for society to find solutions to ensure equitable opportunities in an era where machines are replacing humans in various sectors (industrial, military, healthcare, social, etc.). There is an urgent need to create relevant legislation that ensures respect for workers’ rights. The widespread use of AI requests an analysis of its impact on the labor market [25].

Equity

AI technologies promise to enhance healthcare through improved diagnosis, treatment, and patient outcomes. However, access to these technologies is uneven, increasing existing health disparities. Despite the lower number of healthcare professionals in rural areas, these regions can increasingly benefit from AI to fill gaps in healthcare. AI technologies can provide essential support through remote diagnoses, treatment recommendations, and health monitoring, minimizing staff shortages. However, these AI solutions (easily available in large urban centers) may remain inaccessible to rural areas due to a lack of funding or infrastructure needed to support these technologies, leading to a lower quality of care and delayed diagnoses for rural patients [26]. Likewise, patients with lower incomes might have trouble affording advanced AI treatments or may not have the digital tools, like high-speed internet, needed to use telemedicine and AI services, which can make health inequalities even worse [26].

These ethical questions are not the only ones, but they are the ones that generate the most debate in society. Addressing these ethical concerns requires a holistic response from society, involving AI creators, regulators, and AI users, to establish ethical guidelines and responsible practices in the field of AI and ML [13].

Ethical considerations regarding the use of ML in medicine

There are various ethical issues associated with the use of ML in medicine. They can be classified according to their dimension, namely into epistemic, normative, and overarching ethical problems [27].

Epistemic Considerations

Epistemic considerations refer to knowledge, understanding, and the ways in which we acquire or justify that knowledge. In the context of AI algorithms and technologies, epistemic concerns generally refer to questions about the nature, origin, and validity of the knowledge generated or used by these technologies [27,28]. In this specific context, epistemic concerns can influence trust in automated decisions and the way in which the results are communicated to users and society [27,28]. It is mandatory for society to find satisfactory answers to epistemic concerns to ensure an ethical and responsible application of technology. The epistemic considerations are the inconclusive evidence, the inscrutable evidence, and the misguided evidence [29]. Inconclusive evidence refers to results or evidence that are not conclusive or definitive due to uncertainty or lack of information. Considering devices for heart rate identification used in medicine but also accessible to the general population (such as smartwatches), AI algorithms aim to “diagnose” a patient with an arrhythmia, but without a “clinical eye” to validate arrhythmia, it could simply be due to a defect in the watch or because the 'norm' is inadequately calibrated for that individual (e.g., normal rhythm changes in athletes) [27,29]. The algorithmic results (classification as arrhythmic or not) are probabilistic and not infallible, and therefore, they are rarely sufficient to affirm the existence of a causal relationship (inconclusive evidence) [27,29]. The inscrutable evidence refers to results or evidence whose operating logic cannot be easily understood or explained by humans due to the complexity of the algorithms or the lack of transparency in AI decision-making processes [14,15]. Considering clinical decision support systems (with growing implementation in hospitals and primary care levels), these systems generate treatment recommendations based on collected data. These recommendations, inferred by AI algorithms, may not be understood by healthcare professionals; they do not understand the criteria and logical reasoning followed by the AI to reach a particular conclusion [6,29]. In this situation, there is a risk of inappropriate data use for a particular individual (the data input for decision-making may not be the most suitable for a specific individual), leading to excessive, insufficient, or incorrect prescriptions (due to data bias, as previously discussed) [12,13]. As healthcare professionals rarely have full oversight of the data used to train or test an algorithm, the conclusions (evidence) obtained by the AI are inscrutable evidence [30]. Finally, misleading evidence refers to results or evidence that are incorrect or inaccurate due to errors in the algorithms, input data, or other aspects of the analysis process conducted by AI. Giving an example, one of the most widely used diagnostic software in Oncology (IBM® Watson for Oncology) aims to diagnose neoplasms through image recognition, using a database of images collected from Western populations [31,32]. Widely used in some Eastern countries, such as China, its validity has been questioned. Numerous concerns and doubts have arisen because the software, originally loaded with a database featuring characteristics of the Western population, leads to issues of concordance and inferior outcomes for Chinese patients compared to their Western countries [31,32]. Algorithmic results can only be as reliable (but also as neutral) as the data on which they are based (misleading evidence).

Normative Considerations

Normative considerations refer to the ethical principles and guidelines that guide the development, implementation, and use of AI in healthcare [16]. These considerations address ethical and moral issues to ensure that AI is applied in a fair, safe, and responsible manner, avoiding unfair outcomes and transformative effects [13,33]. An unfair outcome refers to decisions or actions produced by algorithms that have a negative or discriminatory impact on certain groups of people, often due to biases in the training data or the algorithms themselves [12,13,34]. An action can have a greater impact (positive or negative) when results are assessed on a population scale rather than an individual one. In these cases, the algorithm will “learn” to prioritize groups of patients for whom better outcomes for a specific disease are predicted, at the expense of individual patients (discriminatory effect): AI “accepts” a negative outcome for an individual if it leads to positive outcomes at the group level. Thus, algorithmic decisions may arise that harm an individual of a race or ethnicity different from the group in which they are included [34]. Transformative effects are deep changes that result from the application of AI in different areas of society, leading to improvements in efficiency, automation of tasks, creation of new business models, and transformations in industry and the economy, among other aspects. These transformative effects of AI have the potential to deeply alter the way we live, work, and interact with the world [27]. However, algorithmic activities can reorganize reality in unexpected ways. In the health sector, health apps on mobile phones are increasingly common. People using these apps do not have supervision (or will have limited supervision) over which data can be collected by the app. These data will serve to make recommendations for improving health; however, the absence of supervision limits the ability to contest any recommendations made and results in a loss of autonomy in decision-making, potentially transforming our health and well-being negatively [33,34].

Overarching Considerations

Finally, overarching considerations refer to comprehensive and global principles or guidelines that guide the development, use, and regulation of AI. These principles seek to ensure that AI is developed and applied ethically, responsibly, and in compliance with fundamental values such as transparency, fairness, privacy, and security [27,35]. These overarching principles aim to provide a broad framework for addressing complex ethical issues related to AI. In the context of AI ethics in healthcare, overarching considerations can address important themes that have widespread implications and are not restricted to specific cases; from this perspective, the lack of traceability in medical decision-making algorithms is a crucial point of concern [35]. Traceability refers to the ability to track and understand the decisions and actions taken by AI systems and involves the capacity to understand how an algorithm arrived at a particular conclusion or recommendation [27]. Traceability is crucial for ensuring the transparency, accountability, and fairness of AI systems, allowing developers and users to understand and assess their functioning and impact [36]. Furthermore, traceability is essential for identifying and correcting biases, errors, and unintended consequences of AI systems, promoting an ethical and responsible implementation of the technology [14,18,33]. Traceability is based on five pillars: moral responsibility, shared (or distributed) responsibility, automation bias, safety and resilience, and ethical auditing. Moral responsibility refers to the obligation of developers, users, and owners of AI systems to take responsibility for the ethical consequences of AI algorithm decisions. Automation bias refers to the tendency of people to excessively trust automated systems without questioning or verifying the obtained conclusions. This can lead to undesirable or unfair outcomes, especially when AI systems are influenced by biases (algorithmic biases or flaws). Traceability guarantees that algorithms are safe (protecting the data that compose them, but also the people as “end users” of the algorithmic decisions) and resilient (able to handle adverse or unexpected situations without compromising their functionality or safety). Conducting ethical audits ensures that AI systems operate according to ethical principles and acceptable behavioral standards [36]. Within these traceability concepts, we can infer that, as it is difficult to ascertain damage caused solely by algorithmic activity (detecting the cause of damage), it also becomes difficult to identify who should be held accountable for this damage. If a decision made by an algorithm results in a negative outcome for an individual, in the current legal landscape, the healthcare professional will bear this responsibility. This legal framework complicates the implementation of future prevention mechanisms since it is virtually impossible to identify which step of the algorithm was responsible for the decision that led to a negative outcome. This inability to trace “downstream” in the algorithm the steps that led to an unfavorable or incorrect decision also blocks the development of future protection and surveillance mechanisms that could minimize these situations [33,35,36].

The growing use of AI in healthcare (and more specifically, ML algorithms) could have long-term effects on society beyond just medicine. One particular concern is that doctors might lose important skills if they rely too much on AI for diagnoses and treatment plans, potentially becoming less capable of making these decisions on their own [37]. There are also economic worries, such as smaller healthcare providers struggling to keep up with larger ones that can afford advanced AI, which might lead to more industry consolidation. Additionally, there is a risk that society could become too dependent on AI, which could cause major problems if the technology fails or is unavailable during events like cyberattacks [37]. As AI systems become more advanced and capable of making independent decisions, questions arise about their moral and ethical status. AI has an increasing role in critical areas like medicine, sparking debates about responsibility and rights. For instance, if an AI system performs surgery with minimal human involvement and an error occurs, it’s unclear who should be held accountable: the AI, its developers, or the healthcare providers. Similarly, if AI is used to make ethical decisions, such as prioritizing patients for organ transplants, the moral implications are significant, especially when these decisions are made by a machine rather than a human. There is also a theoretical debate about whether highly advanced AI should be granted certain rights, raising deep ethical questions about AI's future role in society [37,38].

## Conclusions

The integration of AI (and its autonomous learning processes or ML) in medicine has revolutionized the global health landscape, providing faster and more accurate diagnoses, personalized treatment, and efficient management of clinical information. However, this transformation is not without ethical challenges that require a comprehensive and responsible approach.

There are various ethical issues raised using AI in medicine. These include general ethical issues such as data bias, lack of transparency, the “black box phenomenon,” and technological unemployment, among others. Only a global and holistic approach to these issues will allow for the establishment of ethical guidelines and responsible practices. Furthermore, concerning AI’s autonomous learning algorithms (i.e., medical decision algorithms), more specific ethical issues arise in the medical field, notably epistemic issues (which focus on the origin and validity of the knowledge generated by AI), normative issues (which address ethical principles to ensure fair and safe application of AI for all), and issues concerning traceability (where moral responsibility for damage caused by algorithmic activities is difficult to determine). Addressing these issues is crucial to ensure that the use of AI in healthcare is implemented ethically and responsibly, providing benefits to populations without compromising fundamental values. Ongoing dialogues among all professionals and regulators involved in the AI industry are of major importance for the safe application of AI in medical practice.

## References

[1] Farhud DD, Zokaei S (2021). Ethical issues of artificial intelligence in medicine and healthcare. Iran J Public Health.

[2] Amisha Amisha, Malik P, Pathania M, Rathaur VK (2019). Overview of artificial intelligence in medicine. J Family Med Prim Care.

[3] Alowais SA, Alghamdi SS, Alsuhebany N (2023). Revolutionizing healthcare: the role of artificial intelligence in clinical practice. BMC Med Educ.

[4] Rashidi HH, Tran N, Albahra S, Dang LT (2021). Machine learning in health care and laboratory medicine: general overview of supervised learning and Auto-ML. Int J Lab Hematol.

[5] Durán JM, Jongsma KR (2021). Who is afraid of black box algorithms? On the epistemological and ethical basis of trust in medical AI. J Med Ethics.

[6] Alsuliman T, Humaidan D, Sliman L (2020). Machine learning and artificial intelligence in the service of medicine: necessity or potentiality?. Curr Res Transl Med.

[7] Deo RC (2015). Machine learning in medicine. Circulation.

[8] Matsuo Y, LeCun Y, Sahani M (2022). Deep learning, reinforcement learning, and world models. Neural Netw.

[9] Lo Vercio L, Amador K, Bannister JJ (2020). Supervised machine learning tools: a tutorial for clinicians. J Neural Eng.

[10] Barata C, Rotemberg V, Codella NC (2023). A reinforcement learning model for AI-based decision support in skin cancer. Nat Med.

[11] Tang L, Li J, Fantus S (2023). Medical artificial intelligence ethics: a systematic review of empirical studies. Digit Health.

[12] Stanfill MH, Marc DT (2019). Health information management: implications of artificial intelligence on healthcare data and information management. Yearb Med Inform.

[13] MacIntyre MR, Cockerill RG, Mirza OF, Appel JM (2023). Ethical considerations for the use of artificial intelligence in medical decision-making capacity assessments. Psychiatry Res.

[14] Kiseleva A, Kotzinos D, De Hert P (2022). Transparency of AI in healthcare as a multilayered system of accountabilities: between legal requirements and technical limitations. Front Artif Intell.

[15] Nolan P (2023). Artificial intelligence in medicine - is too much transparency a good thing?. Med Leg J.

[16] Cannarsa M (2021). Ethics guidelines for trustworthy AI. The Cambridge Handbook of Lawyering in the Digital Age.

[17] Adadi A, Berrada M (2018). Peeking inside the black-box: a survey on explainable artificial intelligence (XAI). IEEE Access.

[18] Smith MJ, Bean S (2019). AI and ethics in medical radiation sciences. J Med Imaging Radiat Sci.

[19] Cath C (2018). Governing artificial intelligence: ethical, legal and technical opportunities and challenges. Philos Trans A Math Phys Eng Sci.

[20] Khalid N, Qayyum A, Bilal M, Al-Fuqaha A, Qadir J (2023). Privacy-preserving artificial intelligence in healthcare: techniques and applications. Comput Biol Med.

[21] Regulation (EU) 2016/679 of the European Parliament and of the Council of 27 April 2016 on the protection of natural persons with regard to the processing of personal data and on the free movement of such data, and repealing Directive 95/46/EC (General Data Protection Regulation). Official Journal of the European Union.

[22] Henz P (2021). Ethical and legal responsibility for artificial intelligence. Discov Artif Intell.

[23] Bleher H, Braun M (2022). Diffused responsibility: attributions of responsibility in the use of AI-driven clinical decision support systems. AI Ethics.

[24] Federspiel F, Mitchell R, Asokan A, Umana C, McCoy D (2023). Threats by artificial intelligence to human health and human existence. BMJ Glob Health.

[25] Walusiak-Skorupa J, Kaczmarek P, Wiszniewska M (2023). Artificial intelligence and employee's health - new challenges [Article in Polish]. Med Pr.

[26] Gurevich E, El Hassan B, El Morr C (2023). Equity within AI systems: what can health leaders expect?. Healthc Manage Forum.

[27] Morley J, Machado CC, Burr C, Cowls J, Joshi I, Taddeo M, Floridi L (2020). The ethics of AI in health care: a mapping review. Soc Sci Med.

[28] Azzam T (2023). Artificial intelligence and validity. New Dir Eval.

[29] Bent B, Goldstein BA, Kibbe WA, Dunn JP (2020). Investigating sources of inaccuracy in wearable optical heart rate sensors. NPJ Digit Med.

[30] Emanuel EJ, Wachter RM (2019). Artificial intelligence in health care: will the value match the hype?. JAMA.

[31] Liu C, Liu X, Wu F, Xie M, Feng Y, Hu C (2018). Using artificial intelligence (Watson for Oncology) for treatment recommendations amongst Chinese patients with lung cancer: feasibility study. J Med Internet Res.

[32] Liu W, Shi X, Lu Z, Wang L, Zhang K, Zhang X (2018). Review and approval of medical devices in China: changes and reform. J Biomed Mater Res B Appl Biomater.

[33] Kleinpeter E. (2017). Four ethical Issues of “e-health”. IRBM.

[34] Garattini C, Raffle J, Aisyah DN, Sartain F, Kozlakidis Z (2019). Big data analytics, infectious diseases and associated ethical impacts. Philos Technol.

[35] Søvik AO (2022). What overarching ethical principle should a superintelligent AI follow?. AI Soc.

[36] Racine E, Boehlen W, Sample M (2019). Healthcare uses of artificial intelligence: challenges and opportunities for growth. Healthc Manage Forum.

[37] Bajwa J, Munir U, Nori A, Williams B (2021). Artificial intelligence in healthcare: transforming the practice of medicine. Future Healthc J.

[38] Zhang J, Zhang ZM (2023). Ethics and governance of trustworthy medical artificial intelligence. BMC Med Inform Decis Mak.

